# Hepatitis Rebound after Infection with Yellow Fever Virus

**DOI:** 10.3201/eid2506.190069

**Published:** 2019-06

**Authors:** Blandine Denis, David Chirio, Diane Ponscarme, Ségolène Brichler, Nathalie Colin de Verdière, François Simon, Jean-Michel Molina

**Affiliations:** Hôpital Saint Louis, Paris, France (B. Denis, D. Chirio, D. Ponscarme, N. Colin de Verdière, F. Simon, J.-M. Molina);; Hôpital Avicenne, Bobigny, France (S. Brichler); Université Paris Diderot, Sorbonne Cité, Paris (F. Simon, J.-M. Molina)

**Keywords:** Yellow fever, persistent hepatitis, Brazil, viruses, France, hepatitis

## Abstract

In 2018, yellow fever with hepatitis was diagnosed for 2 unvaccinated travelers returning to France from Brazil. Hepatitis persisted for >6 months; liver enzyme levels again increased 2 months after disease onset with no detection of yellow fever virus RNA or other pathogens. Persistent hepatitis with hepatic cytolysis rebound probably resulted from immune response.

Although most cases of yellow fever are asymptomatic, some lead to severe liver disease, caused by liver cell cytolysis. In February 2018, yellow fever with hepatitis was diagnosed for 2 patients returning to France after travel to Brazil (Rio de Janeiro coastal area, including the island of Ilha Grande). Both were previously healthy, HIV-negative men, 38 (patient A) and 28 (patient B) years of age; neither had been vaccinated against yellow fever. Their first signs and symptoms occurred 7 (patient B) and 8 (patient A) days after arrival in Brazil, and they sought care in France on day 7 of symptom onset.

Each patient had fever, jaundice, asthenia, thrombocytopenia (57,000 [patient A] and 61,000 [patient B] platelets/mm^3^; reference range >150,000 platelets/mm^3^), and hepatitis (alanine aminotransferase [ALT] >5,000 IU/L and aspartate aminotransferase [AST] >3,400 IU/L; reference range <40 IU/L). One patient also had acute renal failure (serum creatinine 170 µmol/L). For both patients, malaria blood smear was negative, IgM against yellow fever virus was detected on 3 evaluations by IgM capture ELISA, and seroconversion was confirmed by detection of yellow fever virus IgG by indirect ELISA at the National Reference Center for Arboviruses (HIA Laveran, Marseille, France). Results of testing for other viruses (dengue [4 serotypes]; chikungunya; Zika; hepatitis A, B, C, E; cytomegalovirus; and Epstein-Barr) were negative. The patients showed no sign of bleeding and were discharged 2 (patient B) and 3 (patient A) days after admission, after thrombocytopenia and liver enzyme elevations had improved.

The patients remained asthenic for ≈1 month with no other symptoms. Elevated bilirubin levels persisted 6 weeks after discharge for patient A and 8 weeks for patient B; levels even increased in patient A (up to 164 μmol/L on day 14 of symptom onset). Within 3 weeks, liver cytolysis decreased to <4 times the upper limit of the reference range. However, ≈2 months after symptom onset, liver enzyme levels again increased (ALT >1,000 IU/L) (Figure). For each patient, rebound ALT levels were higher than rebound AST levels (patient A: ALT 1,046, AST 301 IU/L; patient B: ALT 1,410, AST 483 IU/L). During the rebound period, yellow fever RNA detection by PCR remained negative. Conversely, for each patient, yellow fever virus neutralizing activities were detected in plasma by a recently developed highly specific test based on a pseudoviral vector releasing pseudotype yellow fever virus–like particles (*1*). Serum from each patient exhibited 100% inhibitory activity against yellow fever virus particles (*1*). Deep sequencing performed on plasma and urine remained negative for eukaryotic pathogens (*2*). Also negative were serum autoimmune antibodies (antinuclear, anti–smooth muscle, anti–liver-kidney microsomal type 1, and antimitochondrial) and anti–soluble liver antigen. For patient B, liver enzyme levels returned to reference range within 6 months; for patient A, AST returned to reference range within 6 months and ALT within 7 months (Figure). Because the patients were asymptomatic at the time of rebound, we did not perform liver biopsies.

**Figure:**
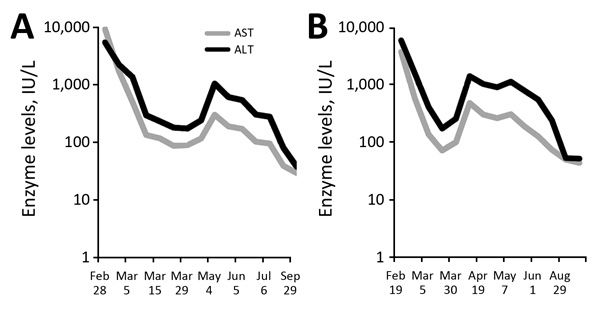
Time course for liver enzyme levels in 2 patients with yellow fever, France, 2018. A) Patient A; B) Patient B; AST, aspartate aminotransferase; ALT, alanine aminotransferase.

Each patient had acquired yellow fever in Ilha Grande, Brazil, as had 11 other nonvaccinated travelers to Brazil in 2018 (*3*), before vaccination against yellow fever was recommended for travelers to the states of São Paulo and Rio de Janeiro. For each of the 2 patients reported here, illness was similar: relatively mild disease despite jaundice and severe cytolysis (AST >1,200 IU/L, ALT >1,500 IU/L at diagnosis), known risk factors associated with higher mortality rates (*4*). For each patient, hepatic cytolysis rebounded ≈2 months after the initial peak, with no clinical signs or symptoms and no detection of other pathogens. Previous reports have described liver cytolysis during yellow fever as being acute, decreasing rapidly on days 9–15, then reaching the upper limits of the reference range on day 16, and some persisting up to 2 months (*5*). Liver biopsy results have shown that liver lesions can persist up to 2 months (*6*). 

We report persistent hepatitis over 6 months with a rebound of hepatic cytolysis after 2 months. In comparison, relapse has been described for ≈3% of patients with hepatitis A (*7*) with a biphasic peak of ALT (*7**,**8*) and detection of hepatitis A virus RNA in plasma (*9*), both 4–8 weeks after the first peak.

In terms of the cause of the hepatic cytolysis rebound in the 2 patients reported here, neither had taken a hepatotoxic drug during the rebound period, and results of deep sequencing for eukaryotic pathogens and autoimmune antibodies were negative. However, major yellow fever virus neutralizing activities were detected. Thus, the rebound could be attributed to host immune response to yellow fever virus rather than to another pathogen or to direct effects of the virus itself. In summary, our results show that yellow fever can induce persistent hepatitis with a rebound of liver cytolysis, probably an immune response to the yellow fever virus.
